# Home Confinement in Previously Active Older Adults: A Cross-Sectional Analysis of Physical Fitness and Physical Activity Behavior and Their Relationship With Depressive Symptoms

**DOI:** 10.3389/fpsyg.2021.643832

**Published:** 2021-05-20

**Authors:** Joana Carvalho, Flávia Borges-Machado, Andreia N. Pizarro, Lucimere Bohn, Duarte Barros

**Affiliations:** Faculty of Sports, Research Centre in Physical Activity, Health and Leisure, University of Porto, Porto, Portugal

**Keywords:** depression, mental health, COVID-19, exercise, multicomponent training

## Abstract

**Aim:**

The aim of our study was to analyze physical activity levels, sitting time, physical fitness, and their relationship with depressive symptoms after home confinement in previously active older adults.

**Methods:**

This cross-sectional study sample comprised 68 older adults (74.24 ± 5.67 years) from a community-based exercise program conducted in Porto, Portugal. After home confinement, participants were assessed in person for lower-body strength (30-s chair stand test), cardiorespiratory fitness (6-min walking test), agility/dynamic balance (8-ft up-and-go test), handgrip strength, and anthropometry. Telephone interviews were performed to evaluate depressive symptoms with the Geriatric Depression Scale – 15 items (GDS-15) and physical activity levels through the International Physical Activity Questionnaire (IPAQ-SV). Individuals were also asked to self-report changes in their physical activity levels and time spent sitting.

**Results:**

Ninety percent of older adults self-reported a decrease in overall physical activity levels, and nearly 65% increased daily sitting time during the home confinement. However, previously active older adults still presented high levels of physical fitness (scores above 50th compared with Portuguese normative values) after 11 weeks of home confinement. Overall, 52.9% of participants scored 5 or more points on GDS-15, which is suggestive of depression. Higher levels of moderate-to-vigorous physical activity (MVPA) and cardiorespiratory fitness were found in the non-depressed group compared with the depressed group. Finally, results from multiple regression analysis revealed that MVPA was negatively associated with depression. This model explained 16.4% of the variability seen in depression score, controlled for age, gender, and education.

**Conclusion:**

Even reporting a decline in physical activity, older adults who previously participated in a formal exercise program, still presented high levels of physical fitness after 11 weeks of home confinement. However, MVPA, but not physical fitness, seems to be an associated depression score in previously active older adults. These results reinforce the importance of older adults to remain physically active, since higher levels of MVPA may have a protective effect on depressive symptoms and, therefore, mitigate the negative impact of home confinement on mental health. Future longitudinal research studies are needed to ascertain these results.

## Introduction

Currently, the number of new COVID-19 cases are on the rise worldwide. Most of the countries already adopted measures of social distance to contain the spread of the virus and even more strict measures like partial or full lockdown to relief their highly pressured health systems (Han et al., 2020).

This unprecedented scenario drastically changed daily routines and restricted individuals’ freedom. In addition, the uncertainty around the pandemic progression, its economic, social, and public health impact, will undoubtedly contribute to emotional distress and increased risk for psychiatric illness among the general population (Pfefferbaum and North, 2020). In line, a recent systematic review found that the COVID-19 pandemic is associated with highly significant levels of psychological distress among the general population (Xiong et al., 2020). Indeed, these findings on mental health are clear and concerning, and can be explained by the fear of being infected or infecting others, mandatory home confinement, and the impossibility to spend time with loved ones that are dying or even for not being allowed to perform the usual farewell rituals. These factors can lead to negative feelings of anxiety, helplessness, depression, irritability, and anger (Mari and Oquendo, 2020). Shortly after the beginning of this pandemic, Holmes et al. (2020) published a call for action in *Lancet Psychiatry* highlighting the need to investigate the impact of COVID-19 on mental health, particularly, on vulnerable groups like older adults, in order to design strategies that mitigate its negative health consequences (Holmes et al., 2020).

Older adults are a vulnerable group that was particularly affected because they were asked to self-isolate at home and avoid any unnecessary contact with other people. While isolated at home, they are likely to have restricted access to physical activity and, therefore, prone to become more sedentary and less active (Valenzuela et al., 2020; Woods et al., 2020), which can lead to consequences such as functional decline, frailty, depression, metabolic syndrome, and increased risk of all-cause mortality (Roshanaei-Moghaddam et al., 2009; Rezende et al., 2014). Concomitantly, social isolation and loneliness are major risk factors linked with poor physical and mental health status (Wu, 2020). These aggregated factors can lead to an unprecedented negative cycle with impact on older adult’s health and well-being.

Mental health problems are common in older adults, particularly depressive symptoms. In Portugal, the estimated prevalence of anxiety and depression are 9.6 and 11.8%, respectively (de Sousa et al., 2017). These numbers are expected to rise as a result of this health crisis. Krendl and Perry (2020) found that older adults reported higher depression and greater loneliness succeeding the onset of the pandemic (Krendl and Perry, 2020). Adding to this, Nguyen et al. verified that people aged 60 years or above had increased the likelihood of depression and poor health-related quality of life during the ongoing pandemic (Nguyen et al., 2020).

The relationship between physical activity and depression has been described. Physical exercise, a subcategory of physical activity, can positively impact several biological (from inflammation to the endocrine system) and psychosocial processes like social support and self-esteem that integrate the pathophysiology of depression (Kandola et al., 2019). Robust evidence from a meta-analysis of prospective cohort studies found a protective role of physical activity in the incidence of depression among older adults, since individuals with high levels of physical activity had 21% lower odds of depression compared with those with low levels of physical activity (Schuch et al., 2018). Moreover, low levels of physical activity and sedentary behavior (SB) are associated with a greater risk of depression and mental health problems (Mammen and Faulkner, 2013; Huang et al., 2020). SB has been associated with negative physical and mental health consequences, independent of the physical activity (Smith et al., 2018). The results, so far, indicate an association between SB and perceived stress, risk of insomnia and sleep disturbance, cognitive performance, and dementia (Falck et al., 2017; Yang et al., 2017; Ashdown-Franks et al., 2018), which are critical problems among older adults. However, some results are still unclear and inconsistent, and need further investigation.

Also, there seems to be an important association between physical fitness and depression. Another meta-analysis found that low cardiorespiratory fitness was associated with a 64% higher risk of depression when compared with high cardiorespiratory fitness; however, authors reinforced the substantial heterogeneity seen between studies (Kandola et al., 2019). In this sense, it is expected that physically active older adults might have increased protection against the detrimental impact of home confinement on mental health (Caputo and Reichert, 2020; Moreira et al., 2020).

The impact of home confinement caused by the COVID-19 outbreak on older adults’ activity behaviors and mental health has yet to be investigated in depth, as this information can enlighten policy efforts during the next few months of this pandemic. Therefore, this study aims to analyze physical activity levels, sitting time, and physical fitness status and their relationship with depressive symptoms after home confinement in older adults that used to participate in formal exercise program.

## Materials and Methods

A cross-sectional study was conducted on post-confinement data aiming to analyze physical activity levels, sitting time, physical fitness, and their relationship with depressive symptoms in previously active older adults.

### Sample

Participants were engaged since October 2019 in a program of physical activity called “Mais Ativos Mais Vividos” (MAMV), which consists of a community-based exercise program hosted at the Faculty of Sports of the University of Porto, Portugal. This scholar year intervention started in the middle of October 2019. Participants maintained their normal in-person exercise sessions until March 18, the day on which the country entered a state of emergency, and all activities ceased.

The eligibility criteria for this study were physically active subjects aged ≥65 enrolled at the exercise program MAMV in the scholar year 2019–2020. Participants who developed any acute health condition (e.g., cardiovascular events and osteoarticular or musculoskeletal injuries/conditions in which exercise is contraindicated) during the home confinement were excluded from this study since that could affect physical performance and testing procedures.

**FIGURE 1:**
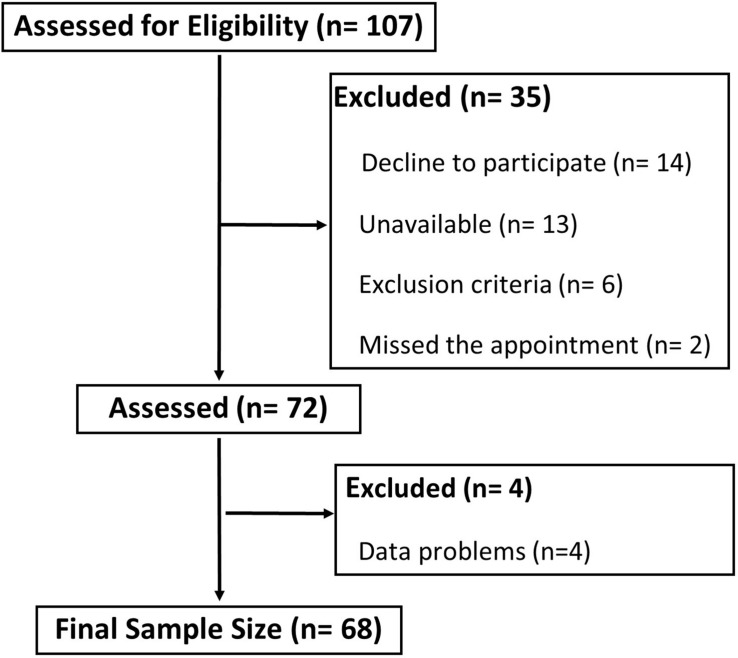
Study flowchart.

Before the COVID-19 pandemic, 107 older adults were participating in the MAMV exercise program. After 11 weeks of home confinement, all participants were contacted, and 72 accepted to participate and were evaluated. From those, 68 (74.24 ± 5.67 years) had valid data (Figure 1). Our sample was composed of participants that were enrolled in this program for at least 3 years (nearly 70% of the participants) for an average of 5 years.

### Instruments

#### Anthropometry

Height (cm) was measured using a calibrated portable stadiometer (Seca 217, Hamburg) with 0.1-cm resolution. Body weight (kg) was measured to the nearest 0.1 kg (InBody Co., Ltd., South Korea). Body mass index (BMI) was calculated as the weight divided by the squared height (in meters). Overweight and obesity were classified according to WHO cutoff points (World Health Organization, 1995).

#### Physical Fitness

Physical fitness was measured using the Senior Fitness Test (SFT) (Rikli and Jones, 2001), a reliable instrument to assess older adults with ≥60 years old. This physical battery includes lower-body strength (30-s chair stand test), cardiorespiratory fitness (6-min walking test), and agility/dynamic balance (8-ft up-and-go test). To measure the lower body strength, participants were asked to sit in a 43-cm armless chair with their arms folded across the chest and execute the maximum of full stands within 30 s. The score was the total number of stands completed within that time. Cardiorespiratory fitness was measured using a 6-min walk test in which participants were asked to walk as fast as possible for 6 min. The score was the total distance walked along a 45.72-m rectangular course, which was marked every 4.57 m. Finally, to assess agility and dynamic balance, participants were asked to stand up, walk an 8-ft distance (2.44 m), turn the cone marker around, and return to the seated position, as quickly as possible. The time necessary to complete this test was registered (in seconds). Participants’ scores were further compared with the normative functional fitness standards for the Portuguese older adults (Marques et al., 2014).

Handgrip strength was obtained with a Jamar Plus + Digital hand dynamometer (Sammons Preston Inc., Bolingbrook, IL, United States) (Mendes et al., 2017). Measurements were carried out following the American Society of Hand Therapists recommendations (American Society Hand Therapy, 1983), and each participant performed three attempts with a pause of 1 min between them. The maximum value of the three measurements with the dominant hand was registered in kilograms force (kgf).

#### Physical Activity

Physical activity was measured using the Portuguese validated short version of the International Physical Activity Questionnaire (IPAQ-SV) (Craig et al., 2003). The IPAQ-SV comprises the frequency and duration of vigorous and moderate physical activity, walking, and one additional item on time spent sitting. Participants were classified into three categories of physical activity: high, moderate, and low, according to IPAQ-SV scoring protocol available at http://www.ipaq.ki.se/ (IPAQ, 2010).

Participants were categorized as high if they performed vigorous-intensity activities on at least three days and accumulated at least 1,500 MET-min/week, or any combination of walking, moderate-intensity, or vigorous-intensity activities on at least 7 days, achieving a minimum of 3,000 MET-min/week. As moderate, if they performed three or more days of vigorous activity of at least 20 min/day, or five or more days of moderate-intensity activity or walking of at least 30 min per day; or five or more days of any combination of walking, moderate-intensity or vigorous-intensity activities achieving a minimum of at least 600 MET-min/week. Finally, participants were categorized as low if they did not meet the criteria for the categories moderate or high.

Additionally, similar to other studies (Cleland et al., 2018), average time spent on moderate to vigorous physical activity (MVPA) was computed as the result of the sum of the number of days with vigorous activity multiplied by the time spent in this intensity, plus the number of days with moderate activity multiplied by the time spent in this intensity, and then divided by 7 days. The questionnaire was applied in the form of an interview via phone calls.

#### Changes in Movement Behaviors

Participants were asked to self-report changes in physical activity and sitting time through two short answer questions: in comparison with your prior confinement routine, did your daily physical activity/sitting time increased, maintained, or decreased during home confinement? (DGS, 2020).

#### Depression

The Geriatric Depression Scale – 15 items (GDS-15) is a widely used self-report measure to screen depressive symptoms among older adults. Of the 15 items, 10 indicate the presence of depression when answered positively, while the question numbers 1, 5, 7, 11, and 13 indicate depression when answered negatively (Pocklington et al., 2016). A score ≥5 points is suggestive of depression (Pocklington et al., 2016). The validated Portuguese version present a good internal consistency (α Cronbach = 0.83) (Apóstolo et al., 2014). This evaluation was based in interviews, via telephone.

### Procedures

The previous in-person MAMV exercise program encompassed a multicomponent training (MT), twice a week, in 60-min sessions. Sessions comprised 10 min of warm-up, 30 min of aerobic and resistance training, 10 min of balance and coordination exercises, and a cool down of approximately 10 min. Warm-up included mobility/stretching and low-intensity aerobic exercises such as walking. Afterward aerobic exercises at moderate-to-vigorous intensity (such as dance, step, and circuit training were performed. Leg press, squats, chest press, vertical row, core exercises, biceps’ curl, and arm raises are some examples of the resistance training exercises aiming at major muscle groups (two sets of 10–12 repetitions at moderate intensity). Balance and coordination exercises will gradually reduce base support and include dynamic movements. The cool down was used for some respiratory and stretching/flexibility exercises.

The in-person exercise program ceased on March 18, the day on which the Portuguese government declared the state of emergency and mandatory lockdown. Therefore, very strict measures of physical–social distancing were adopted to control the outbreak. People were not allowed to circulate in public spaces or roads except to obtain essential goods and services, work, or go for short walks, alone or with members of the same household, following the safety and physical distance guidelines.

#### Data Collection

On June 1, mandatory confinement ended, and it was allowed to gather under 20 persons outdoors following the safety and physical distance guidelines. In the following days, participants from the MAMV program were contacted twice by phone. In the first contact, they were invited to participate in in-person evaluations of anthropometry (e.g., height and body weight) and physical fitness (e.g., SFT and handgrip strength). For those who agreed to participate, the in-person evaluations were scheduled.

The in-person evaluations were carried out in the outdoor park at the Faculty of Sports, University of Porto. Two participants were scheduled every 20 min to avoid potential contact between individuals. Facial masks were mandatory throughout all procedures, and safety guidelines were strictly followed. Afterward, a second contact was made to apply physical activity (e.g., IPAQ-SV and self-reported changes in movement behaviors) and depression questionnaires (e.g., GDS-15). Five phone call attempts were made, and those who did not answer were excluded.

All participants signed an informed consent form, and all procedures were conducted in full accordance with the Helsinki Declaration. The study was approved by the Ethical Committee of the Faculty of Sports of the University of Porto (Ref CEFADE15.2020).

### Statistical Analyses

Data normality was verified using Kolmogorov–Smirnov tests. Measures of central tendency [mean and median (Mdn)] and dispersion [standard deviation and interquartile range (IR)] were used as appropriate to describe sample characteristics. Between-group comparisons were performed with both parametric (independent *t*-test) and non-parametric approaches (Mann–Whitney *U* test) for continuous variables and chi-squared test for categorical variables. Univariable linear regression models were performed to examine associations between the independent variables (i.e., MVPA, sitting time, and physical fitness variables) and depression score (continuous variable). Multivariable linear regression models were built by entering those variables that were statistically significant in the univariable analysis controlling for age, gender, and educational level, with enter selection of variables. Multicolinearity was verified using VIF. The significant level was set as 95%, and procedures were carried out with SPSS version 24 (IBM, Chicago, IL, United States).

## Results

### Sample Characteristics

The characteristics of the sample are shown in Table 1. Participants had an average age of 74 years old (range 65–90), were predominantly female (60.3%), and with 6–12 years of education. Moreover, 21 participants (30.9%) completed primary school and 18 (26.5%) had a degree. The majority (73.5%) were classified as overweight or obese. Approximately 90% of older adults self-reported a decrease in overall physical activity levels, while 64.7% increased daily sitting time during the home confinement. Participants report low levels of daily MVPA [13.93 min per day (0.00–30.00)]. Moreover, according to IPAQ-SV, 57.3% were included in the moderate or high category of physical activity.

**TABLE 1:** Characteristics of the study population.

	Overall (*n* = 68)
Age	74.24 ± 5.67
**Gender**	
Female, *n* (%)	41 (60.3)
**Education**	
<6 years, *n* (%)	21 (30.9)
6–12 years, *n* (%)	29 (42.6)
>12 years, *n* (%)	18 (26.5)
BMI, kg/m^2^	27.15 ± 3.48
Overweight and obesity, *n* (%)	50 (73.5)
**Physical activity and sitting time**	
MVPA, min/day	13.93 [0.00–30.00]
Daily sitting time, min/day	300 [180–480]
Physical activity status	
Low PA, *n* (%)	29 (42.7)
Moderate PA, *n* (%)	28 (41.2)
High PA, *n* (%)	11 (16.1)
**Changes in PA during confinement**	
Decreased, *n* (%)	61 (89.7)
Maintained, *n* (%)	4 (5.9)
Increased, *n* (%)	3 (4.4)
Changes in daily sitting time	
Decreased, *n* (%)	2 (2.9)
Maintained, *n* (%)	22 (32.4)
Increased, *n* (%)	44 (64.7)
**Physical fitness**	
Cardiorespiratory fitness (m)	565.22 ± 82.48
Lower body strength (reps)	21.10 ± 4.22
Agility (s)	5.04 ± 1.02
Handgrip strength (kgf)	28.93 ± 9.15
Depression (score)	4.81 ± 2.63
Symptoms of depression, *n* (%)	36 (52.9)

*Values are mean ± SD (continuous variables), median [interquartile range] or percentage (categorical variables). PA, physical activity; MVPA, moderate to vigorous physical activity; BMI, body mass index.*

Participants presented high levels of fitness on lower body strength (above the 90th percentile), on agility/dynamic balance (above the 75th percentile), and on cardiorespiratory fitness test (above the 50th percentile) when comparing with the Portuguese population functional fitness standards on the 75- to 79-year-old age group.

Participants presented an average GDS-15 score of 4.81 ± 2.63 points. Two groups were created based on the GDS-15 score: non-depressed group comprising participants with scores <5 and the depressed group for older adults who scored ≥5 points. Thus, 52.9% of the participants (*n* = 35) had suggestive symptoms of depression or indicative symptoms of depression (i.e., GDS > 10, *n* = 1).

### Comparisons Between Non-depressed and Depressed Groups on Physical Fitness, Physical Activity, and Sitting Time

No significant differences were registered between groups for sitting time (*p* > 0.05). Additionally, MVPA was significantly higher within the non-depressed group [Mdn 25.71 (5.89–120)] compared with those in the depressed group [Mdn 4.29 (0–18.75), *p* = 0.029) (Table 2). Concomitantly, most participants from the depressed group were categorized as low physical activity levels on IPAQ-SV, whereas the majority of the non-depressed group were classified as moderate or high physical activity level (*p* < 0.001). No significant differences between groups were found for handgrip strength, lower-body strength, agility/dynamic balance, or cardiorespiratory fitness (all *p* > 0.05). After controlling for age, gender, and educational level, group differences were sustained for MVPA (data not shown).

**TABLE 2:** Comparisons between the non-depressed and depressed group.

	Non-depressed group (*n* = 32)	Depressed (*n* = 36)	Statistical inference
Age	73.56 ± 6.21	74.89 ± 5.16	*t*(66) = −0.962, *p* = 0.340
Gender			
Female, *n* (%)	21 (65.6)	20 (55.6)	χ^2^(1) = 0.718, *p* = 0.397
**Physical activity and sitting time**			
MVPA (min/day)	25.71 [5.89–120]	4.29 [0–18.75]	*U* = 308.05, *p* = 0.001
Daily sitting time (min/day)	300 [180–345]	360 [195–585]	*U* = 668, *p* = 0.255
**Physical activity status**			
Low PA, *n* (%)	7 (21.9)	22 (61.1)	χ^2^(2) = 18.588, *p* < 0.001
Moderate PA, *n* (%)	14 (43.8)	14 (38.9)	
High PA, *n* (%)	11 (34.4)	0 (0)	
**Changes in PA during confinement**			
Decreased, *n* (%)	27 (84.4)	34 (94.4)	χ^2^(2) = 1.908, *p* = 0.385
Maintained, *n* (%)	3 (9.4)	1 (2.8)	
Increased, *n* (%)	2 (6.2)	1 (2.8)	
**Changes in daily sitting time**			
Decreased, *n* (%)	2 (6.2)	0 (0)	χ^2^(2) = 2.592, *p* = 0.274
Maintained, *n* (%)	11 (34.4)	11 (30.6)	
Increased, *n* (%)	19 (59.4)	25 (69.4)	
**Physical fitness**			
Cardiorespiratory fitness (m)	584.79 ± 92.59	543.77 ± 66.68	*t*(63) = 1.862, *p* = 0.067
Lower body strength (reps)	20.61 ± 3.73	21.53 ± 4.61	*t*(65) = −0.884, *p* = 0.380
Agility (s)	5.03 ± 1.13	5.04 ± 0.92	*t*(66) = −0.034, *p* = 0.973
Handgrip strength (kgf)	28.18 ± 8.49	29.59 ± 9.77	*t*(66) = −0.633, *p* = 0.529

*Values are mean ± SD (continuous variables), median [interquartile range], or percentage (categorical variables). PA, physical activity; MVPA, moderate-to-vigorous physical activity; BMI, body mass index; *t*, independent sample *t*-test; χ^2^, Chi square test; *U*, Mann–Whitney *U* test.*

### Relationship Between Physical Activity, Sitting Time, and Physical Fitness With Depression Score

Linear regression analysis was conducted to explore the association between MVPA, sitting time, and physical fitness variables with depression score (continuous scale). Results from the crude analysis showed that MVPA [*R*^2^ = 0.191; *F* (1,63) = 15.538; *p* < 0.001] and sitting time [*R*^2^ = 0.067; *F* (1,66) = 4.759; *p* = 0.033] was associated with GDS-15 score. Physical fitness variables [cardiorespiratory fitness (*p* = 0.141), agility (*p* = 0.977), handgrip strength (*p* = 0.891), and lower body strength (*p* = 0.592)] were not associated with GDS-15 scores (Table 3). In multivariable analyses, after adjusting the model for age, gender, and education level, MVPA remained significantly associated with GDS-15 score (*B* = −0.019, 95% CI: −0.029 to −0.009, *p* < 0.001), whereas sitting time did not (*p* = 0.054). The whole model explained 16.4% of the variance seen in depression score (Table 3).

**TABLE 3:** Associations between moderate-to-vigorous physical activity, sitting time, and physical fitness with depression score.

Parameters	Unstandardized coefficients		95% IC	*p*-value
	
	*B*	SE		
**MVPA model**				
*Unadjusted MVPA model (R^2^ = 0.191)*				
MVPA	−0.019	0.005	[−0.029 to −0.010]	<0.001
**Adjusted MVPA model (*R*^2^ = 0.164)**				
MVPA	−0.019	0.005	[−0.029 to −0.010]	<0.001
Gender	0.095	0.602	[−0.827 to 1.793]	0.875
Age	0.071	0.053	[−0.070 to 0.167]	0.184
Education	0.091	0.395	[−0.933 to 0.777]	0.819
**Sitting time model**				
*Unadjusted sitting time model (R^2^ = 0.067)*				
Sitting time	0.003	0.002	[0.001 to 0.007]	0.033
**Adjusted sitting time model (*R*^2^ = 0.028)**				
Sitting time	0.003	0.002	[0.001 to 0.007]	0.054
Gender	0.216	0.649	[−1.082 to 1.513]	0.741
Age	0.062	0.057	[−0.052 to 0.175]	0.281
Education	0.112	0.435	[−0.758 to 0.982]	0.798
**Physical fitness models**				
Unadjusted cardiorespiratory fitness (*R*^2^ = 0.034)	−0.006	0.004	[−0.014 to 0.002]	0.141
Unadjusted lower body strength (*R*^2^ = 0.004)	0.042	0.078	[−0.113 to 0.197]	0.592
Unadjusted hand grip strength (*R*^2^ < 0.001)	0.005	0.035	[−0.066 to 0.076]	0.891
Unadjusted agility (*R*^2^ < 0.001)	−0.009	0.318	[−0.645 to 0.626]	0.977

## Discussion

The present study analyzed the physical activity levels, sitting time, and physical fitness status after 11 weeks of home confinement due to the COVID-19 pandemic and explored their relationship with depressive symptoms on older adults that were previously regular participants of a formal exercise program. Overall, participants self-reported drastic changes in physical activity and sitting time, but presented high physical fitness levels after 11 weeks of home confinement, above the average for the Portuguese normative values (Marques et al., 2014). Symptomatology of depression was found in 52.9% of previously physically active older adults after confinement. Our data showed significant differences between groups as the non-depressed group presented higher values of MVPA compared with the depressed group. Furthermore, only MVPA remained negatively associated with GDS-15 scores in the regression analysis.

Depression affects more than 264 million people worldwide, independently of age, gender, and ethnic background. This highly prevalent mental disorder is one of the leading causes of disability and a major contributor to the overall global burden of disease (World Health Organization, 2020a). The long-term mental health impact of COVID-19 may take weeks, months, or even years to become evident (Rajkumar, 2020). According to the World Health Organization, older adults may become more anxious, angry, stressed, agitated, and withdrawn during the outbreak or while in quarantine – particularly those isolated or with impaired cognitive function (World Health Organization, 2020b).

It is well known that PA, and particularly exercise, have a therapeutic effect on individuals’ physical and mental health, especially when performed outdoors or in groups (Burtscher et al., 2020). However, our data showed that being previously active for an average of 5 years did not prevent older adults (52.9%) from reporting suggestive symptoms of depression. Despite not measuring if the participants were already experiencing this symptomatology before the home confinement, this finding was somewhat unexpected for two reasons: First, given the well-known therapeutic effects that PA, and particularly exercise, have on individuals’ physical and mental health, especially when performed outdoors or in groups, as our program did (Burtscher et al., 2020), and second, because some reviews elicit the long-lasting effect of light-to-vigorous exercise on depressive symptoms (Helgadóttir et al., 2017), and only 11 weeks had passed between ceasing the exercise program and the post-confinement assessments. Nevertheless, findings in this matter are still controversial and inconsistent as a systematic review and meta-analysis of randomized controlled trials showed little evidence of a long-term beneficial effect of exercise in patients with clinical depression (Krogh et al., 2011) and suggested a short-term effect instead.

Several authors (Valenzuela et al., 2020; Woods et al., 2020) highlighted the severe consequences of physical–social distancing measures not only in mental health but also on the physical health of older populations. For example, the study results of Ammar et al. (2020) revealed a decline in all levels of PA during the home confinement period and a far more significant increase on sitting time. Our participants reported similar results as almost 90% of the sample decreased their usual PA levels, and 65% increased sitting time, therefore, changing drastically their usual behaviors with possible negative health consequences. The results on PA levels were expected because our sample was previously highly active older adults who performed exercise sessions at least twice a week. However, the results in sitting time may not truly represent their time spent on sedentary activities since it is well known that older adults tend to overestimate their PA levels, particularly MVPA, and underestimate the time spent on SB (Grimm et al., 2012; Harvey et al., 2013). Our non-significant difference on SB between groups of depression can be due to the differences among sedentary activities since a very recent study found that mentally passive SBs, such as watching television, could increase the risk of depression, whereas mentally activity sedentary activities were not associated (Huang et al., 2020). Therefore, more research on sedentary patterns is needed, particularly in these strange times. Nevertheless, and despite recommendations that home confinement should not preclude people from being physically active (Ammar et al., 2020; Centers for Disease Control and Prevention, 2020; Sepúlveda-Loyola et al., 2020), only 57.4% of our sample kept moderate-to-high levels of PA during confinement. In fact, 78.2% of the non-depressed participants were in the moderate and high PA group, while more than half of the participants with depression (61.1%) reported low PA levels.

Moderate-to-vigorous physical activity was negatively associated with GSD-15 score, explaining 16.4% of the variance of GDS-15 score, adjusted for age, gender, and education. Similarly, results of Callow et al. (2020) from a multiple regression model including total physical activity, age, sex, and education accounted for 7.2% of the variance in total depression scores; however, depression symptoms were measured using a 30-item GDS. In agreement, Carriedo et al. (2020), in their cross-sectional study conducted through an online questionnaire, emphasized that older adults who regularly engaged in MVPA during confinement reported lower scores in depressive symptoms. The potential role of physical activity in mitigating depressive symptoms in older adults seems in line with other studies (Kandola et al., 2019) conducted before the COVID-19 pandemic. Emerging research on the efficacy of home-based exercise training programs to maintain and improve PA levels and physical fitness during home confinement is now becoming available (Marcos-Pardo et al., 2020) and could be a way to mitigate the negative mental health impact of confinement. Moreover, many of the adopted strategies for exercise during the COVID-19 pandemic had involved technologies and virtual meetings reducing the possible negative effects of social distancing and isolation, keeping people active and in touch with each other (Armitage and Nellums, 2020; Burtscher et al., 2020; Jiménez-Pavón et al., 2020).

Regarding physical fitness, our participants were above the 50th percentile for the Portuguese population on cardiorespiratory fitness, and above the 75th and 90th percentile on the 8-ft up-and-go test and 30-s chair stand test, respectively (Marques et al., 2014). Therefore, as opposed to the expected, individuals presented higher levels of physical fitness after an 11-week home confinement period. Moreover, none of the physical fitness tests showed significant differences between groups. These results contrast with other investigations conducted either in the older or in younger groups. Rantanen et al. (2000) found that depressed mood was associated with increased risk of steep handgrip strength decline in older men. In another investigation, the results showed that women with depressive or anxiety disorders had significantly lower grip strength compared with healthy controls, but this association was not found in men (van Milligen et al., 2011). Research has also been pointing toward a significant inverse relationship between cardiorespiratory fitness and depressive scores (Galper et al., 2006) independent of physical activity levels and suggesting that exercise interventions aiming at aerobic endurance may play an important role in preventing depression in older adults (Yamagata et al., 2013). In contrast to our investigation, Lee (2015) divided 173 older women (65–80 years) into two groups (GDS-K score ≥ 14 and GDS-K score < 14) and found significant differences between the two groups in the 6-min walk and also in other physical performance tests. The author suggests that improvements in physical fitness through exercise can improve depression by helping the recovery of self-confidence or inducing emotional responses in older adults (Lee, 2015). A possible explanation for this lack of association in our study could be attributed to our sample having above-average levels of physical fitness contrasting to usual research that is conducted in non-exercisers. Nonetheless, the relationship between physical fitness and depressive symptoms remains partially unclear, and there is a need for additional research to clarify if a causal relationship exists.

In short, our study results reinforce the importance to remain active during home confinement due to the potential of MVPA in mitigating the negative impact on depressive symptoms. Further evidence is necessary to acknowledge the role of physical fitness components and SB patterns on preventing or mitigating depressive symptoms.

This cross-sectional study has some strengths that must be addressed. Several authors highlighted the need for research on mental health consequences of these unprecedented times on the general population (Callow et al., 2020; Carriedo et al., 2020; Moreira et al., 2020; Rajkumar, 2020; Torales et al., 2020) and specifically on vulnerable and older populations (Troutman-Jordan and Kazemi, 2020; Vahia et al., 2020), whether through online surveys (3), editorials or opinion papers (2), and review articles (2). Nevertheless, to the best of our knowledge, to date, the current study is the first to present results from in-person physical performance tests and telephone evaluations with previously physically active older adults.

Additionally, it is important to acknowledge some limitations. First, data on PA and depression were gathered via an interview by phone call, which could have a potential influence on our results. Second, self-reported PA assessment has well-known limitations in older adults. Furthermore, the cross-sectional nature of this study does not allow us to determine a cause-and-effect relationship between MVPA and depression score, and our small sample size limits the generalization of our results.

In summary, our findings suggest that MVPA is negatively associated with depressive scores in a home confinement situation. We considered that our findings can be used as a starting point to manage isolation restrictions more effectively and to develop strategies to promote physical activity among older adults during situations of forced lockdowns, as in the present COVID-19 pandemic. Therefore, a multidisciplinary team involving psychologists, social workers, and PA professionals should work together to create strategies to promote at-home physical activity and mental well-being programs during this pandemic as a key to limit depressive symptoms.

## Data Availability Statement

The datasets used and/or analyzed during the current study are available from the corresponding author on reasonable request.

## Ethics Statement

The studies involving human participants were reviewed and approved by Ethics Committee of the Faculty of Sports, University of Porto (CEFAD 15.2020). The patients/participants provided their written informed consent to participate in this study.

## Author Contributions

All authors listed have made a substantial, direct and intellectual contribution to the work, and approved it for publication.

## Conflict of Interest

The authors declare that the research was conducted in the absence of any commercial or financial relationships that could be construed as a potential conflict of interest.
